# 培美曲塞联合顺铂对比吉西他滨联合顺铂一线治疗晚期非鳞非小细胞肺癌的随机、对照、多中心临床研究

**DOI:** 10.3779/j.issn.1009-3419.2012.10.03

**Published:** 2012-10-20

**Authors:** 岩 黄, 云鹏 刘, 建英 周, 农 徐, 宝兰 李, 钢 伍, 健 方, 凯 李, 晓睛 刘, 巍 刘, 铀 卢, 孟昭 王, 文超 刘, 后杰 梁, 沂平 张, 诚 黄, 顺金 王, 雅杰 王, 世英 于, 建华 常, 哲海 王, 志皇 胡, 力 张

**Affiliations:** 1 510060 广州，中山大学肿瘤防治中心 Department of Medical Oncology, Sun Yat-sen University Cancer Center, 510060 Guangzhou, China; 2 510060 广州，华南肿瘤学国家重点实验室 State Key Laboratory of Oncology in South China, 510060 Guangzhou, China; 3 110001 沈阳，中国医科大学附属第一医院 Department of Medical Oncology, First Hospital of China Medical University, 110001 Shenyang, China; 4 310003 杭州，浙江大学医学院附属第一医院 Department of Respiratory Diseases, First Affiliated Hospital of Medical School of Zhejiang University, 310003 Hangzhou, China; 5 Department of Chemotherapy, the First Affiliated Hospital of Medical School of Zhejiang University, 310003 Hangzhou, China; 6 101149 北京，北京市胸科医院 Department of General Medicine, Beijing Chest Hospital, Capital Medical Hospital, 101149 Beijing, China; 7 430022 武汉，华中科技大学同济医学院附属协和医院 Tumor Center, Union Hospital, Tongji Medical College, Huazhong University of Science and Technology, 430022 Wuhan, China; 8 100142 北京，北京大学肿瘤医院 Department of Thoracic Medical Oncology, Peking University School of Oncology, Beijing Cancer Hospital and Institute, 100142 Beijing, China; 9 300060 天津，津市肿瘤医院 Department of Thoracic Medical Oncology, Tianjin Medical University Cancer Institute and Hospital, 300060 Tianjin, China; 10 100071 北京，中国人民解放军307医院 Department of Thoracic Medical Oncology, Affiliated Hospital of Academy of Military Medical Sciences, 100071 Beijing, China; 11 050011 石家庄，河北医科大学第四医院 Department of Medical Oncology, Hebei Medical University Fourth Hospital, 050011 Shijiazhuang, China; 12 610041 成都，四川大学华西医院 Department of Thoracic oncology, West China Hospital, West China School of Medicine Sichuan University, 610041 Chengdu, China; 13 100730 北京，中国医学科学院协和医院 Department of Respiratory Diseases, Peking Union Medical College Hospital, Peking Union Medical College and Chinese Academy of Medical Science, 100730 Beijing, China; 14 710032 西安，第四军医大学西京医院 Department of Oncology, Xijing Hospital, Fourth Military Medical University, 710032 Xi'an, China; 15 400038 重庆，第三军医大学第一附属医院 Department of Oncology, First Affiliated Hospital, Third Military Medical University, 400038 Chongqing, China; 16 310022 杭州，浙江省肿瘤医院 Department of Medical Oncology, Zhejiang Cancer Hospital, 310022 Hangzhou, China; 17 350014 福州，福建省肿瘤医院 Department of Thoracic Oncology, Fujian Provincial Cancer Hospital, 350014 Fuzhou, China; 18 330006 南昌，南昌大学附属第二医院 Department of Oncology, Second Affiliated Hospital of Nanchang University, 330006 Nanchang, China; 19 200433 上海，上海市长海医院 Department of Oncology, Changhai Hospital of Shanghai, Second Military Medical University, 200433 Shanghai, China; 20 430030 武汉，华中科技大学同济医学院附属同济医院 Cancer Center, Tongji Hospital, Tongji Medical College, Huazhong University of Science and Technology, 430030 Wuhan, China; 21 200032 上海，复旦大学附属肿瘤医院 Department of Medical Oncology, Fudan University Shanghai Cancer Center, 200032 Shanghai, China; 22 250117 济南，山东省肿瘤医院 Department of Medical Oncology, Shandong Cancer Hospital, 250117 Jinan, China

**Keywords:** 肺肿瘤, 培美曲塞, 吉西他滨, 顺铂, 化学疗法, Lung neoplasm, Pemetrexed, Gemcitabine, Cisplatin, Chemotherapy

## Abstract

**背景与目的:**

目前晚期非小细胞肺癌（non-small cell lung cancer, NSCLC）的标准治疗仍是含铂两药方案，国内外多项临床研究显示培美曲塞联合顺铂在晚期非鳞NSCLC具有较好的疗效及安全性，本研究拟评估国产培美曲塞二钠联合顺铂一线治疗晚期NSCLC的疗效与安全性。

**方法:**

本研究是一项多中心、随机、阳性药物平行对照的临床试验。入组患者按1:1随机分为两组，分别接受培美曲塞二钠联合顺铂（PC组）或吉西他滨联合顺铂（GC组）治疗，21天为一个周期。主要研究终点为无进展生存期，次要研究终点主要包括1年生存率、客观缓解率、无3度或4度毒性生存期及其安全性。

**结果:**

全国20家研究中心共纳入288例患者（各组144例），基于全分析集进行分析，PC组与GC组患者的中位无进展生存期分别为168天（5.6个月）和140天（4.7个月）（*P*=0.16）；1年生存率分别为50.0%和54.9%（*P*=0.47）；客观缓解率分别为24.4%和14.2%（*P*=0.06）；无3度/4度毒性生存期分别为11.3个月和8.1个月（*P*=0.23）。总体不良反应发生率PC组明显低于GC组（81.95% *vs* 93.75%, *P*=0.003）。

**结论:**

两种含铂方案治疗晚期非鳞NSCLC具有相似的疗效，但PC方案不良反应更轻，有望成为晚期非鳞NSCLC一线治疗的新选择。

## Abstract (en)

**Background and objective:**

Platinum-based doublet chemotherapy is still the standard first-line treatment for non-small cell lung cancer (NSCLC). Previous studies have demonstrated that pemetrexed combined with platinum had promising efficacy and safety profile in NSCLC, especially in patients with nonsquamous NSCLC. This trial was conducted to evaluate the efficacy and safety of pemetrexed made in China as first-line treatment.

**Methods:**

The present study was a randomized, controlled, multicenter clinical trial. Patients were randomly assigned (1:1) to receive cisplatin plus pemetrexed chemotherapy (PC group) or gemcitabine plus cisplatin (GC group) every 3 weeks. The primary end point was progression free survival (PFS) and the secondary end points included 1 year survival rate, objective response rate (ORR), survival without grade 3/4 toxicity (SWT3/4) and safety profile. Results A total of 288 patients from 20 institutions across China were enrolled into the study. Based on the Full Analyses Set (FAS), the PFS was 168 days (5.6 months) *vs* 140 days (4.7 months) (*P*=0.16), one year survival rate was 50.0% *vs* 54.9% (*P*=0.47), ORR was 24.4% *vs* 14.2% (*P*=0.06) in the PC group and the GC group, respectively; Survival without grade 3/4 toxicity was 11.3 months in GC group *vs* 8.1 months in PC group (*P*=0.23). In terms of the safety, side effects were less observed on the PC group (81.95% *vs* 93.75%, *P*=0.003). The main side effects included leukopenia, neutropenia, emesis, anemia, thrombopenia.

**Conclusion:**

The both regimens have similar efficacy as the treatment for advanced nonsquamous NSCLC, but pemetrexed plus cisplatin regimen has better safety profile and seems to have longer PFS, which makes it a new option as the first line setting.

对于不可切除的局部晚期或转移性非小细胞肺癌（non-small cell lung cancer, NSCLC），目前的标准治疗仍为含铂两药方案化疗。被推荐与铂类联合的化疗药主要包括紫杉醇、多西他塞、吉西他滨、长春瑞滨等^[1]^，这些方案一线治疗晚期NSCLC的无进展生存时间（progression free survival, PFS）为4个月-6个月，中位生存期为8个月-10个月^[2-5]^，各方案疗效相似。近年来培美曲塞在NSCLC中的治疗价值得到广泛研究，国外多项研究^[6-8]^显示其在非鳞NSCLC中较吉西他滨方案具有更好的疗效及安全性，因而美国FDA已批准其与铂类联合用于晚期非鳞NSCLC的一线治疗。

为确定国产培美曲塞（普来乐^®^，江苏豪森药业）联合顺铂治疗晚期NSCLC的临床疗效和不良反应，按照国家食品药品监督管理局的要求，本研究组于2009年8月-2010年9月在全国20家研究中心开展该试验，选择初诊的晚期非鳞NSCLC患者随机分为两组，分别接受国产培美曲塞联合顺铂和吉西他滨联合顺铂方案治疗，并观察两组的疗效和不良反应。

## 1 对象与方法

### 1.1 纳入标准

入选的患者必须符合以下标准：组织学确诊的局部晚期或转移非鳞NSCLC（腺癌+大细胞癌），且不适合根治性治疗；患者至少有1个可测量病灶存在；按照治疗计划患者需要接受一线化疗；若患者曾接受过放疗，其放疗范围必须 < 25%骨髓区域，且未采用过全骨盆或胸部照射；既往的放疗或手术至少已经结束4周；ECOG为0分-2分；预期生存时间至少12周；依从性良好；各器官功能水平适合化疗；患者签署知情同意书；年龄满18周岁以上，75岁以下。

### 1.2 排除标准

若符合以下任何一条，患者将被排除：明确或怀疑有脑转移的患者；同时接受其他任何抗肿瘤治疗；活动性感染；根据研究者的判断，有严重的危害患者安全、或影响患者完成研究的伴随疾病；妊娠或哺乳期妇女；既往有明确的神经或精神障碍史，包括癫痫或痴呆；以前有其它的恶性疾病，但宫颈原位癌及基底细胞癌除外；无法按要求接受化疗前预处理；未控制的第三间隙积液；合并有常见毒性反应标准（Common Terminology Criteria for Adverse Events, CTCAE）3.0版中的3度或4度外周神经病变。

### 1.3 治疗方法

两治疗组患者分别接受如下治疗。试验组（Pemetrexed+Cisplatin, PC）：培美曲塞二钠500 mg/m^2^，d1，静滴时间 > 10 min；顺铂75 mg/m^2^静滴30 min-120 min；该组患者还需接受标准的叶酸补充、维生素B12补充及地塞米松预处理。对照组（Gemcitabine+Cisplatin, GC）：吉西他滨1, 000 mg/m^2^，d1、d8，30 min内静滴；顺铂75 mg/m^2^静滴30 min-120 min。两治疗方案每3周为1疗程（最多6程）直至肿瘤进展或不可耐受的不良反应。

### 1.4 疗效及不良反应评价

根据实体瘤疗效评价标准1.1（Response Evaluation Criteria in Solid Tumors, RECIST）及CTCAE 3.0标准对疗效及不良反应进行评估，将疗效分为完全缓解（complete response, CR）、部分缓解（partial response, PR）、疾病稳定（stable disease, SD）和疾病进展（progressive disease, PD）。基线时在给药前4周内进行肿瘤测量，给药后每2疗程重复一次，直至肿瘤进展或患者退出临床试验。在研究结束前需记录所有不良事件的发生情况，不论其是否与研究药物有因果关系。

### 1.5 随访和生存分析

患者完成研究治疗后均将进入随访期。对于结束研究治疗而尚未进展的患者，要求其每（6±1）周返院复查肿瘤情况，直至肿瘤进展或死亡；对于疾病进展及因各种原因退出研究的患者，对其每3个月进行一次生存随访，直至患者死亡或失访。生存随访由各研究单位采用门诊或电话方式进行，末次随访时间为2012年4月30日。

### 1.6 统计方法

试验样本量的确定严格遵照国家相应法规执行（每组至少100例）。试验结果采用统计产品与服务解决方案（SPSS 17.0）软件进行分析。根据意向治疗原则，疗效分析将在所有接受至少一次剂量治疗的患者中进行。使用*Kaplan-Meier*方法评价时间相关事件的分布情况并生成相应曲线，主要研究终点PFS通过*Fisher’s*双侧精确检验进行比较。*Cox*风险比例模型用来对预后因子进行校正后的两组疗效比较。两组不良反应的比较使用*Fisher’s*精确法检验。*P* < 0.05为差异具有统计学意义。

## 2 结果

### 2.1 入组情况

全国20家单位共入组300例患者并接受随机分配，其中288例接受治疗（两组各144例），治疗后脱落14例（4.5%），剔除3例（1.04%）。两组患者中至少有一次疗效评价的病例数均为127例，无疗效评价（34例）的主要原因有：不良事件/不可耐受毒副反应（19例）、患者要求退出（10例）、失访（3例）、筛选失败和疾病进展各1例。基线期两组患者在性别、年龄、PS评分、组织学类型、疾病分期、既往治疗史以及既往疾病史等各指标的差异均无统计学意义（表 1），两组均衡可比。

**1:** 接受治疗的患者基线临床特征的比较 Baseline Patient and Disease Characteristics for Patients received study treatment

Variable	Pemetrexed/Cisplatin (*n*=144)	Gemcitabine/Cisplatin (*n*=144)	*P*
Age (yr)			0.99
Median (Range)	55 (27-77)	56 (27-73)	
Gender			0.24
Male	84 (58.3%)	73 (50.7%)	
Female	60 (41.7%)	71 (49.3%)	
PS status^*^			0.13
0	25 (17.4%)	34 (23.8%)	
1	111 (77.1%)	104 (72.7%)	
2	8 (5.5%)	5 (3.5%)	
Histology^*^			0.50
Adenocarcinoma	140 (97.2%)	142 (99.3%)	
Large cell	3 (2.1%)	1 (0.7%)	
Mixed	1 (0.7%)	0	
Staging^*^			0.19
Ⅲb	26 (18.1%)	17 (11.9%)	
Ⅳ	118 (81.9%)	126 (88.1%)	
Adjuvant chemo history^*^			0.77
No	139 (96.5%)	137 (95.8%)	
Yes	5 (3.5%)	6 (4.2%)	
Radiotherapy history			0.99
No	136 (94.4%)	137 (95.1%)	
Yes	8 (5.6%)	7 (4.9%)	
Surgical history			0.40
No	108 (75.0%)	115 (79.9%)	
Yes	36 (25.0%)	29 (20.1%)	
Concurrent disease			0.23
No	89 (61.8%)	78 (54.2%)	
Yes	55 (38.2%)	66 (45.8%)	
^*^Partial clinical data of a patient in GC group were missed. PS: performance status.			

### 2.2 疗效分析

PFS为该研究的主要研究终点。基于全分析数据集（full analysis set, FAS）进行分析，试验组与对照组各有17例、13例数据删失，中位PFS分别为168天（5.6个月）和140天（4.7个月），两组差异无统计学意义（HR=0.84, 95%CI: 0.66-1.07, *P*=0.16）。两组患者的PFS曲线见图 1。对性别、年龄、疾病分期和PS评分等因素进行亚组分析，结果显示各亚组中两治疗方案的PFS差异仍无统计学意义，见图 2。

**1:**
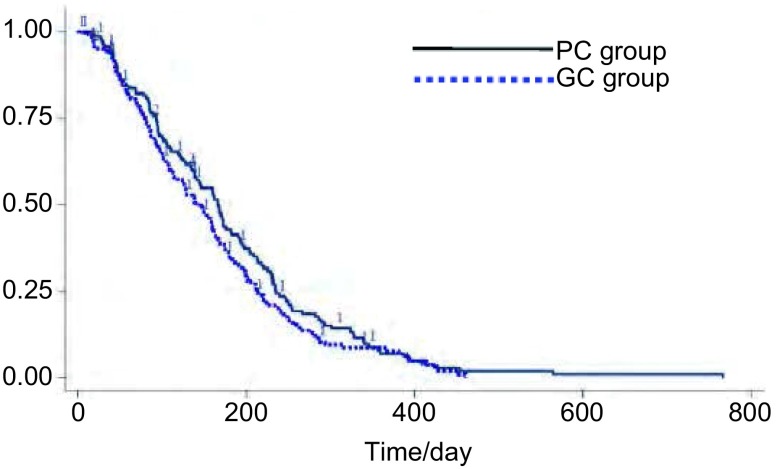
两组患者无进展生存时间的生存曲线 The PFS curves of patients in both groups. PFS: Progression free survival; PC: Pemetrexed+Cisplatin; GC: Gemcitabine+ Cisplatin

**2:**
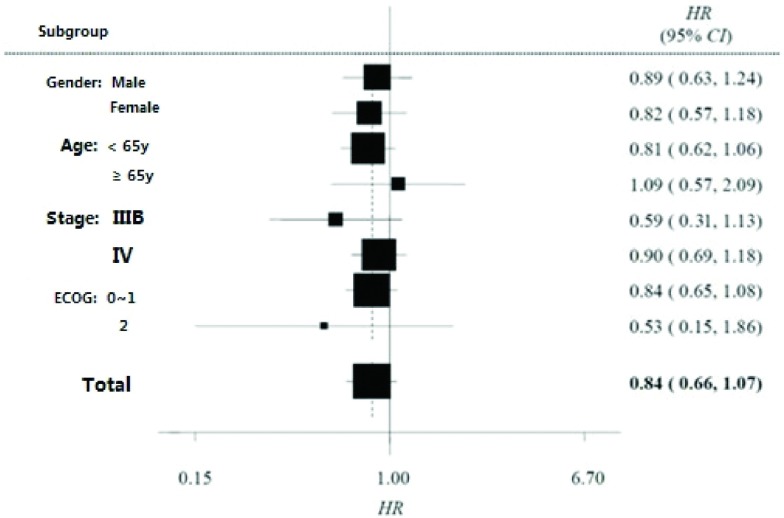
无进展生存期亚组分析森林图 PFS hazard ratios (PC over GC) in subgroups according to baseline characteristics

该研究的次要疗效终点包括1年生存率、客观缓解率（objective response rate, ORR）、无毒性生存期（survival without toxicity, SWT）等。试验组与对照组的1年生存率分别为50%（72/144）和54.9%（79/144），差异无统计学意义（*P*=0.48）；客观缓解率分别为24.4%（31/127，CR 1例、PR 30例、SD 80例、PD 14例）和14.2%（18/127，CR 0例、PR 18例、SD 92例、PD 17例），差异无统计学意义（*P*=0.06）；无3度、4度毒性生存期（SWT3/4）是指从随机分组的日期到首次记录任何一种3度/4度毒性或死亡的时间，其既可评估治疗手段为患者带来的生存获益，亦能反映治疗为患者所带来的风险（严重毒性）。试验组与对照组的中位SWT3/4分别为335天（11.2个月）和243天（8.1个月）（*P*=0.23），而SWT4则分别为518天（17.3个月）和407天（13.6个月）（*P*=0.36），见图 3。

**3:**
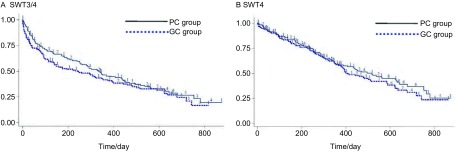
两组患者无度毒性生存期比较。A：无3/4度毒性生存期；B：无4度毒性生存期 The survival without toxicity (SWT) between groups. A: SWT grade 3/4; B: SWT grade 4

### 2.3 安全性分析

接受治疗的288例患者均进行了安全性分析。两组各有118例（82.0%）、135例（93.8%）患者发生了不良反应，试验组总体不良反应发生率低于对照组，差异有统计学意义（*P*=0.003）。试验组和对照组各有5例受试者发生了严重不良事件，发生率分别为3.5%和3.7%，差异无统计学意义。在所有不良反应中，只有血小板减少的发生率在两组间具有统计学差异。不良反应发生情况见表 2。

**2:** 两组患者主要不良反应情况 Main adverse events in both groups

Aderse events	Overall incidence (%)		*P*	Moderate or severe incidence (%)		*P*
	PC group	GC group		PC group	GC group	
Leukopenia	43.75	47.92	0.55	4.17	6.25	0.60
Neutropenia	44.44	40.97	0.63	13.19	15.28	0.74
Anaemia	23.61	33.33	0.09	1.39	3.47	0.45
Thrombocytopenia	7.64	18.75	0.001	1.39	9.03	0.01
Erythrocytopenia	11.81	17.36	0.24	0.00	2.08	0.25
Vomitting	33.33	41.67	0.18	4.17	6.94	0.44
Nausea	32.64	37.50	0.46	1.39	2.78	0.68
Anorexia	15.28	16.67	0.87	1.39	0.69	099
Elevated ALT	4.86	7.64	0.47	1.39	0.69	0.99
Fatigue	7.64	5.56	0.64	0.69	0.69	0.99

## 3 讨论

大多数肺癌患者在诊断时已是局部晚期或出现远处转移，故总体5年生存率仅有15.9%^[9]^，其治疗目的为延长患者生存期及提高生活质量。目前含铂两药方案治疗晚期NSCLC已达疗效瓶颈^[3-5]^，总体有效率约为25%-35%，中位生存期为8个月-10个月，而且由于较重的不良反应，此类方案的应用受到一定限制。因此有必要探索新的有效药物治疗晚期NSCLC。

培美曲塞是新一代多靶点的抗叶酸药物，最初由美国礼来公司作为抗代谢类抗癌药进行研究和开发。该药物对核酸合成过程中的多个重要酶具有很强的抑制作用，尤其对胸膜间皮瘤有突出疗效^[10]^，并率先取得该药在胸膜间皮瘤中的适应症。2004年Hanna等^[7]^发表的研究证实培美曲塞在二线治疗NSCLC时较多西他塞具有更好的安全性，而在腺癌患者中的疗效更佳。2008年Scagliotti等^[6]^又发表了培美曲塞联合顺铂一线治疗晚期NSCLC的研究，结果显示培美曲塞联合顺铂方案较对照组的安全性更好，而腺癌患者（*n*=847，总生存期为12.6个月*vs* 10.9个月）和大细胞癌患者（*n*=153，总生存期为10.4个月*vs* 6.7个月）的总生存期较对照组更长。基于这几项研究，美国癌症综合网络（National Comprehensive Cancer Network, NCCN）和美国临床肿瘤学会（American Society of Clinical Oncology, ASCO）等多个临床肿瘤学指南^[1]^均将培美曲塞联合铂类方案作为非鳞NSCLC患者的Ⅰ类推荐方案。

本研究为国产培美曲塞（普来乐^®^）一线适应症的注册临床研究。其设计与国外Scagliotti等的研究设计类似，但我们本次研究纳入的患者均为非鳞NSCLC，且主要研究终点为PFS。根据国家《药品注册管理办法》对临床试验的最低病例数要求，该试验不少于100对，考虑到病例脱落及患者依从性问题，本次试验样本量确定为300例（各150例）。

该试验中位无进展生存期（mPFS）在两组分别为5.6个月和4.7个月，试验组较对照组有延长PFS的趋势，但差异无统计学意义（*P*=0.16）。该研究结果与既往的报道相似，2008年Scagliotti等^[6]^研究显示在非鳞癌患者中PC组与GC组的中位PFS分别为5.3个月和4.7个月，两组PFS差异无统计学意义，结果与本研究基本一致。若以HR=1.2作为非劣效性研究的界值，则该结果可说明非鳞NSCLC患者使用培美曲塞联合顺铂一线治疗的mPFS非劣于吉西他滨联合顺铂。次要研究终点方面，本研究中培美曲塞联合顺铂治疗组的总有效率（25.2%）、PFS（约5.8个月）、1年生存率（50%）与既往报道的结果基本一致^[3, 11, 12]^。此外，本研究还收集了入组患者的肿瘤组织标本共266例，后续我们将对其进行相关的分子标志物检测，以期探索不同方案的优势人群进而实现晚期肺癌的个体化治疗。

无毒性生存期是指从随机化开始到首次出现任何3度/4度毒性或死亡的时间，这一指标可以体现患者从治疗中获益的情况，同时可反映患者接受治疗的毒性风险，对化疗药物在疗效相当的情况下注重了安全性的要求。Scagliotti等^[13]^对其进行的培美曲塞一线治疗NSCLC的研究进行了回顾性分析，结果显示非鳞癌患者在两组的SWT3/4分别是5.9个月和2.8个月（*P* < 0.001），SWT4分别是10.2个月和8.7个月（*P* < 0.001）。本研究结果虽然SWT3/4及SWT4在两组中的差异均无统计学意义，但试验组的SWT都较对照组延长近100天，且其风险比（HR）分别为0.84（95%CI: 0.63-1.12）和0.87（95%CI: 0.63-1.18），与前述结果基本一致，差异无统计学意义可能是因为存在较多删失病例以及样本量较少的缘故。此外，本研究中使用的吉西他滨剂量较Scagliotti等的剂量稍低（后者为1, 250 mg/m^2^），这也可能是对照组SWT较国外研究稍长的一个原因。

本研究中试验组与对照组的不良反应主要包括白细胞减少（43.8% *vs* 47.9%）、中性粒细胞减少（44.4% *vs* 41.0%）、呕吐（33.3% *vs* 41.7%）、恶心（32.64% *vs* 37.50%）、贫血（23.6% *vs* 33.3%）、食欲不振（15.3% *vs* 16.7%）、血小板减少（7.6% *vs* 18.8%）等。血液学毒性方面试验组白细胞减少、贫血、血小板减少的发生率均低于对照组，但除血小板减少的发生率两组有统计学差异（*P*=0.008）外，其余各项试验组和对照组相比差异均无统计学意义；非血液学毒性方面，试验组恶心、呕吐、食欲不振、ALT/AST升高、皮疹、便秘的发生率略低于对照组，但差异均无统计学意义。严重不良事件的发生率在两组中基本一致。本研究总的不良反应发生率与国外研究结果相当^[6, 8]^，但中重度不良反应发生率两组均低于国外研究结果，可能与样本量较少以及不同人种对化疗的耐受程度不同有关，但目前尚无足够研究数据支持后者的假设。

上述结果表明，与对照组治疗方案相比，研究药物培美曲塞二钠联合顺铂一线治疗局部晚期或转移性非鳞NSCLC的疗效与安全性均较好，总体不良反应发生率更低，而血小板减少的发生率明显低于对照组。
